# Amino acid changes during transition to a vegan diet supplemented with fish in healthy humans

**DOI:** 10.1007/s00394-016-1237-6

**Published:** 2016-06-11

**Authors:** Amany Elshorbagy, Fredrik Jernerén, Marianne Basta, Caroline Basta, Cheryl Turner, Maram Khaled, Helga Refsum

**Affiliations:** 1 Department of Physiology, Faculty of Medicine, University of Alexandria, Alexandria, Egypt; 2 Department of Pharmacology, University of Oxford, Oxford, UK; 3 Pain Management Unit, Department of Anaesthesia, Medical Research Institute, University of Alexandria, Alexandria, Egypt; 4 Department of Nutrition, Institute of Basic Medical Sciences, University of Oslo, Oslo, Norway

**Keywords:** Egyptian Orthodox Christians, Body mass index, Lean mass, Branched-chain amino acids, Sulfur amino acids, Mass spectrometry

## Abstract

**Purpose:**

To explore whether changes in dietary protein sources can lower plasma branched-chain amino acids (BCAAs), aromatic amino acids and sulfur amino acids (SAAs) that are often elevated in the obese, insulin-resistant state and in type 2 diabetes.

**Methods:**

Thirty-six subjects (mean age 31 ± 2 years) underwent a voluntary abstinence from meat, poultry, eggs, and dairy products for 6 weeks, while enriching the diet with fish, in fulfillment of a religious fast. Subjects were assessed 1 week before the fast (V1), 1 week after initiation of the fast (V2) and in the last week of the fast (V3). Thirty-four subjects completed all three visits.

**Results:**

Fasting plasma BCAAs decreased at V2 and remained low at V3 (*P* < 0.001 for all). Valine showed the greatest decline, by 20 and 19 % at V2 and V3, respectively. Phenylalanine and tryptophan, but not tyrosine, also decreased at V2 and V3. The two proteinogenic SAAs, methionine and cysteine, remained stable, but the cysteine product, taurine, decreased from 92 ± 7 μmol/L to 66 ± 6 (V2; *P* = 0.003) and 65 ± 6 μmol/L (V3; *P* = 0.003). A progressive decline in plasma glutamic acid, coupled with an increase in glutamine, was observed. Plasma total and LDL cholesterol decreased at V2 and V3 (*P* < 0.001 for all).

**Conclusion:**

Changing dietary protein sources to plant- and fish-based sources in an ad libitum setting lowers the plasma BCAAs that have been linked to diabetes risk. These findings point to habitual diet as a potentially modifiable determinant of fasting plasma BCAA concentrations.

**Electronic supplementary material:**

The online version of this article (doi:10.1007/s00394-016-1237-6) contains supplementary material, which is available to authorized users.

## Introduction

Accumulating evidence suggests that excess intake of animal protein is detrimental to body adiposity and metabolic health. Omnivores have higher 5-year weight gain compared to vegetarians and vegans [1]. Animal protein intake was prospectively associated with risk of obesity in 1750 men, while plant protein was protective [2]. In 38,000 subjects, high animal protein intake predicted twofold higher risk of developing type 2 diabetes [3]. Risk of gestational diabetes [4] and type 2 diabetes in children [5] also increased with high consumption of animal protein. Among the different protein sources, red meat and poultry are the most consistently associated with weight gain, inflammation, impaired glucose metabolism and diabetes [1, 6, 7]. Interventional studies [8, 9], albeit in contrast to epidemiologic findings [10], also implicate dairy products in deterioration of insulin sensitivity. A notable exception to animal protein sources, in relation to adverse outcomes, is fish. Predominant fish eaters gain less weight over time [1, 6], and, at least in populations with prevalent obesity, fish intake is associated with lower incidence of type 2 diabetes [11].

The associations of meat intake with obesity and related morbidity often persist after adjustment for fat intake [2, 3], suggesting that it may be the difference in amino acid composition that explains the associations. Both sulfur amino acids (SAAs) and branched-chain amino acids (BCAAs) are linked to human obesity and insulin resistance. Plasma BCAAs and aromatic AAs are elevated in insulin-resistant obese individuals [12]. In normo-glycemic individuals, high concentrations of leucine, isoleucine, valine, tyrosine and phenylalanine predict future type 2 diabetes [13] and insulin resistance [14]. Methionine, the only essential SAA, is not associated with adiposity, but its products, S-adenosylmethionine (SAM) and cysteine, are [15–17]. Homocysteine and cystathionine are intermediates in the methionine-to-cysteine pathway and not typically ingested in diet. These amino acids show modest inverse and positive associations with adiposity, respectively [17].

Perturbed BCAA metabolism in adipose tissue is thought to partly explain their elevation in obesity, insulin resistance and type 2 diabetes [18, 19]. We sought to explore another possibility, i.e., that BCAA (and SAA) elevation is at least partly attributable to dietary patterns that predispose to insulin resistance. High intake of meat and milk increases fasting plasma BCAAs [8]. Methionine and cysteine are more abundant in animal-derived proteins than in plant proteins [20]. We observed modest positive associations of plasma total cysteine (tCys) with total and animal protein intakes [15].

Despite increasing recognition of amino acids as predictors of metabolic outcomes, little is known about the dietary determinants of the human plasma amino acid profile, and how far it responds to dietary manipulation. We studied the changes in BCAAs and SAAs during a 6-week abstinence from all animal-derived protein except fish.

## Methods

### Subjects

The study was conducted in 36 sedentary young men and women recruited among Egyptian Orthodox Christians during their yearly religious fast. During the fasting period, which lasts 43 days and terminates on the 7th of January, subjects abstain from eating all animal-derived food apart from fish, without overall limitation of food or liquid intake. The fast resembles the Nativity Fast undertaken by Greek Orthodox Christians prior to Christmas [21].

Data and plasma samples were collected on three visits (V1, V2, V3), where V1 was during the week prior to the start of fasting, V2 during the first week of fasting and V3 during the last week of fasting. Exclusion criteria comprised pregnancy, weight loss of >2 kg over the last month, chronic renal or liver insufficiency, regular moderate or strenuous physical exercise, and intake of medication known to affect body composition (e.g., steroids). Thirty-four subjects completed all 3 visits.

All subjects were informed about the nature of the study and signed an informed consent prior to all procedures. The study was approved by the Ethics Committee of the Faculty of Medicine, Alexandria University.

### Data collection

#### Lifestyle and dietary data

Personal and family history of diabetes, hypertension, cardiovascular disease (angina or myocardial infarction), and the use of lipid-lowering drugs, vitamins, or supplements were recorded using self-administered questionnaires. Cigarette smoking was recorded as current, ex-, or never-smoker. Habitual coffee consumption (cups/day) was assessed.

Dietary data were collected using a semiquantitative food frequency questionnaire, which measured the frequency of consumption of animal protein (meat, poultry, fish), in a main meal, during the week preceding each visit. Frequency of consumption of milk and dairy products was collected over the previous 2 days, where one unit of dairy intake represented a cup of milk (at least 150 mL) or a pot of yoghurt or cheese of any quantity. Intake of eggs was noted as the number of eggs ingested over the 2 days preceding the visit. In addition, participants were given a fill-at-home “compliance diary” where they were required to note days in which deviations from the rules of the fast occurred (e.g., eating meat or eggs during the fasting period).

#### Anthropometric parameters and body composition

Height was measured on V1 to the nearest 0.5 cm using a wall-mounted stadiometer. Weight was measured to the nearest 0.1 kg in light outdoor clothing on V1–V3. Body mass index (BMI) was calculated as weight (kg)/height squared (m^2^). Body weight and body composition (lean mass, fat mass, and percent body fat) were measured on V1, V2, and V3 using a whole-body bioelectrical impedance analysis (BIA) analyzer (InBody 220, Biospace, Korea).

### Blood sampling and biochemical assays

#### Blood sampling

Three blood samples were collected from each participant, one on each visit, after an overnight fast. Samples were collected into EDTA-lined vacuum tubes chilled on ice. Immediately after withdrawal, blood was centrifuged for 30 s, and 200 µL of the plasma supernatant added to 600 µL 4 % v/v perchloric acid and re-centrifuged for 2 min. The resultant supernatant was used for assay of SAM and S-adenosylhomocysteine (SAH). The remaining plasma was re-centrifuged for 3 min, and the plasma supernatant was aliquoted and stored at −80 °C until analysis of amino acids and clinical biochemistry parameters.

#### Clinical biochemistry

Plasma concentrations of total cholesterol, LDL cholesterol (LDL-C), HDL-C, triglycerides, total protein, albumin, and glucose levels were measured by calorimetric assays on a Stat Fax 1904 Plus spectrometer (Awareness Technology, Inc., Palm City, Florida, USA), with absorbance at 474–505 nm.

Fasting plasma insulin was measured by an ELISA (Diagnostic Automation/Cortez Diagnostics Inc., Calabasas, CA, USA) according to the manufacturer’s instructions. Insulin resistance was evaluated by the homeostasis model assessment of insulin resistance (HOMA-IR), calculated as: fasting insulin [μU/mL] × [fasting glucose (mg/dL)/18.01]/22.5.

#### Amino acid assays

Amino acids and creatinine were assayed by liquid chromatography-tandem mass spectrometry (LC–MS/MS) using a Prominence LC-20AD XR binary pump (Shimadzu, Kyoto, Japan) coupled to a QTRAP 5500 hybrid triple quadropole mass spectrometer (AB Sciex, Framingham, MA, US). Plasma methionine, tCys, total homocysteine (tHcy), cystathionine, and total glutathione (tGSH) were analyzed in a single run [22]. The protocol [22] was modified to include arginine, valine, proline, leucine, isoleucine, phenylalanine, tyrosine, ornithine, and tryptophan. SAM and SAH were extracted from perchloric acid-treated plasma using the same protocol and conditions as above, adjusted for the dilution of the samples. Taurine, serine, glutamine, glutamic acid, and creatinine were extracted and assayed separately (see Supplementary Method for details). Quantitation of all analytes was based on comparison with standard curves corrected for presence of isotopically labelled internal standards using a 1/x weighting. %CV for all amino acid analyses were ≤5 %, except for SAM, SAH, taurine, tryptophan, GSH and cystathionine, which were <10 %.

### Statistical analysis

The Kolmogorov–Smirnov test was used to determine the distribution of the data. Only a few analytes violated the assumption of normality, and these were log-transformed prior to parametric analysis. Subject characteristics at baseline are presented as mean ± SEM, and men and women were compared by independent samples *t* test or Chi-squared test as appropriate. Diet effects and possible gender differences were analysed using repeated measures ANOVA with inclusion of gender as a between-subjects factor. There was no significant interaction by gender for the majority of factors. Therefore, data were pooled for men and women and presented as gender-adjusted mean ± SEM for the body composition variables, and unadjusted mean ± SEM for the plasma variables. The frequency of consumption of different food items are presented as median (25th, 75th percentile) and compared by Friedman test followed by Wilcoxon signed-rank test for paired measurements. Correlations were assessed by the Spearman rank correlation coefficient. PASW Statistics for Mac (20.0; SPSS Inc., Chicago, IL, USA) and GraphPad Prism (version 6.0f for Mac) were used for analysis and presentation of data. All tests were two tailed and *P* < 0.05 was considered significant.

## Results

### Characteristics of the study population at baseline

Participants included 12 men and 24 women, with a mean age of 29 and 32 years, respectively. Average BMI was well into the overweight range, with 75 % of men and 58 % of women being overweight or obese (Table 1). One-third of subjects reported a family history of type 2 diabetes, and a quarter reported using multivitamins. None of the participants used lipid-lowering drugs or suffered from diabetes or current or previous cardiovascular disease, but two individuals were on antihypertensive medication.

**Table 1:** Characteristics of the study population

	Men (*N* = 12)	Women (*N* = 24)
Age (years)	29.2 (1.6)	32.2 (2.4)
Height (cm)	172 (2.26)	161 (1.37)*
Weight (kg)	85.2 (4.3)	73.4 (3.6)
BMI	29 (1.5)	28.5 (1.6)
Overweight, BMI 25–30 (%)	33	25
Obese, BMI > 30 (%)	42	33
Fat-free mass (kg)	58.4 (2.0)	41.5 (1.3)*
Fat mass (kg)	26.8 (3.1)	32 (2.6)
Body fat (%)	30.6 (2.3)	42.1 (1.6)*
*Lifestyle and medical history*		
Coffee intake (cups/days)	1.3 (0.3)	1.1 (0.2)
Current smokers (%)	8	0
Current or past CVD (%)	8	0
Diabetes mellitus (%)	0	0
Multivitamin users (%)	25	29
Antihypertensive users (%)	8	4
*Family history of disease*		
Type 2 diabetes (%)	33	33
CVD (%)	42	44
*Plasma variables*		
Glucose (mg/dL)	95 (2)	90 (2)
Total cholesterol (mg/dL)	189 (7)	186 (7)
HDL-C (mg/dL)	49.5 (2.9)	57.6 (2.7)
LDL-C (mg/dL)	122 (10)	108 (7)
Triglycerides (mg/dL)	138 (9)	130 (8)
Albumin (g/dL)	4.06 (0.10)	4.04 (0.05)
Total protein (g/dL)	7.09 (0.26)	7.09 (0.14)

Data are presented as mean ± SEM or proportions

*CVD* cardiovascular disease

* *P* < 0.05, independent samples *t* test

### Change in diet after the onset of the fast

**Fig. 1:**
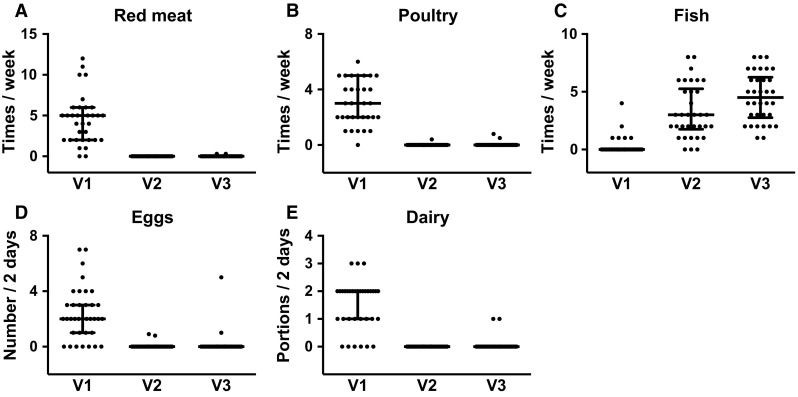
Frequency of consumption of different food groups, presented as number of servings per week (**a**–**c**), or per 2 days (**d**, **e**). Data represent median, 25th–75th percentiles, with individual data plotted. For all food groups, intake at V2 and V3 differed significantly from V1 (*P* < 0.001). Where there is overlap between the percentile lines and most or all data points (e.g., 1-A and 1-B at V2 and V3), individual data dots are obscured by the percentile lines. *V1* baseline, *V2* first week after diet change, *V3* last week of diet change

There were marked changes in the sources of dietary protein after the onset of the fast, with high compliance to abstinence from all animal protein sources apart from fish. Frequency of intake of red meat and poultry in a main meal was relatively high at baseline (median meat and poultry intakes = 5 and 3 times/week, respectively), which is typical in anticipation of the subsequent abstinence, and dropped to zero at V2 and V3 (Fig. 1). On the other hand, 75 % of the population reported no intake of fish in the week preceding the fast, with median frequency of fish intake rising to 3 and 4.5 times per week on V2 and V3, respectively.

### Changes in body composition and clinical biochemistry parameters

A small but significant decrease in BMI was observed at V3, mostly explained by a decline in lean mass (Table 2). Plasma albumin, total protein, and creatinine remained stable throughout the fasting period. There was also no change in total fat mass or body fat %, or in plasma triglycerides. However, plasma total cholesterol decreased by V2, and remained low at V3, almost totally due to a decrease in LDL-C. Neither fasting plasma glucose, nor insulin and HOMA-IR were affected by the diet change (Table 2).

**Table 2:** Effect of the diet on body composition and metabolic parameters

	V1	V2	V3
*Body composition*			
BMI (kg/m^2^)	28.6 (1.3)	28.7 (1.3)	28.2 (1.2)**
Total fat mass (kg)	29.0 (2.2)	28.7 (2.2)	28.8 (2.1)
Fat-free mass (kg)	49.8 (1.2)	50.3 (1.2)	49.1 (1.2)**
Body fat percent	36.1 (1.4)	35.7 (1.4)	36.3 (1.4)
*Plasma metabolic parameters*			
Total cholesterol (mg/dL)	187 (5)	177 (4)**	173 (5)**
HDL-cholesterol (mg/dL)	55.0 (2.3)	55.7 (2.4)	56.6 (2.5)
LDL cholesterol (mg/dL)	113 (6)	103 (5)**	100 (6)**
Triglycerides (mg/dL)	134 (7)	130 (8)	133 (10)
Total protein (g/dL)	7.11 (0.14)	6.97 (0.12)	7.01 (0.14)
Albumin (g/dL)	4.05 (0.05)	4.04 (0.05)	4.04 (0.05)
Creatinine (μmol/L)	54.1 (2.0)	54.1 (2.2)	53.7 (2.1)
Glucose (mg/dL)	91.5 (1.3)	91.8 (2.3)	88.8 (2.6)
Insulin (μU/mL)	7.53 (0.23)	7.60 (0.51)	7.82 (0.36)
HOMA-IR	1.71 (0.06)	1.82 (0.12)	1.74 (0.11)

Data are presented as gender-adjusted mean (SEM) for body composition parameters, and unadjusted mean (SEM) for clinical biochemistry parameters, calculated from repeated measures ANOVA. *N* = 34–36, except for insulin and HOMA-IR measurements, *N* = 27. Where the ANOVA was significant (*P* < 0.05), pairwise comparisons versus V1 were conducted

*V1* baseline, *V2* first week after diet change, *V3* last week of diet change, *HOMA-IR* homeostasis model of insulin resistance

** *P* < 0.001

### Amino acid changes in response to altering dietary protein sources

#### Branched-chain and aromatic amino acids

**Fig. 2:**
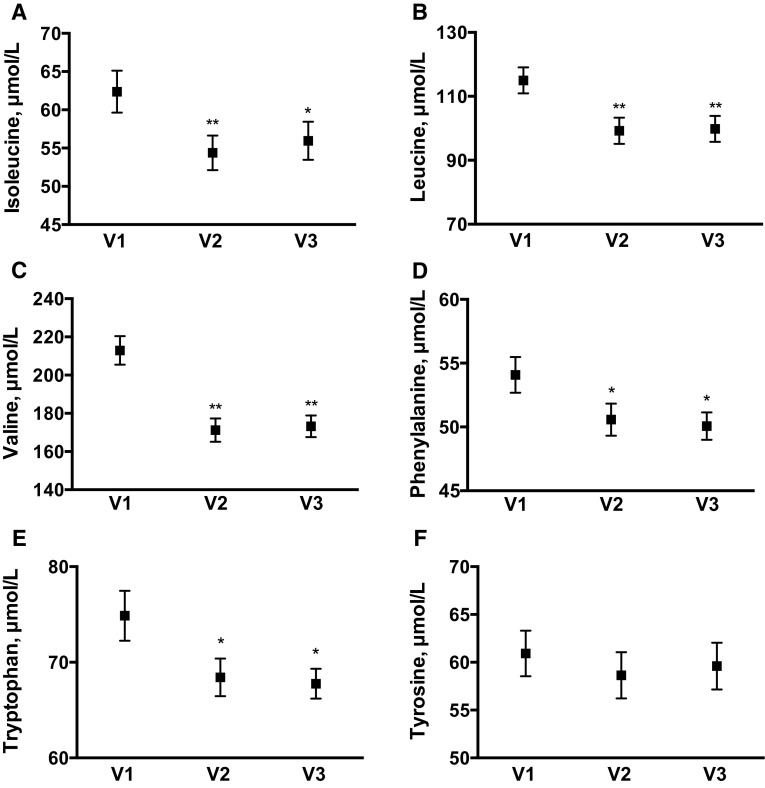
Fasting plasma branched-chain (**a**–**c**) and aromatic (**d**–**f**) amino acids at *V1* (baseline), *V2* (first week after diet change), and *V3* (last week of diet change). Data represent mean (SEM). *P < 0.01; **P < 0.001 for pairwise comparisons versus V1, with repeated measures ANOVA *P* < 0.05

An early and sustained decrease was observed in the plasma concentrations of BCAAs (Fig. 2). Leucine was 14 and 13 % lower at V2 and V3, respectively, versus V1 (*P* < 0.001 for both), while isoleucine decreased by 12 and 10 %. Valine showed a greater decline (by 20 and 19 % at V2 and V3, respectively; both *P* < 0.001). Among the aromatic amino acids, there was no significant effect of the diet on plasma tyrosine concentrations, while phenylalanine and tryptophan were significantly decreased at V2 and V3.

Plasma concentrations of valine, leucine, and isoleucine showed significant positive correlations with HOMA-IR at V2 and V3 (*r* = 0.43–0.50), while the correlations at V1 were in the same direction (*r* = 0.25–0.27) but not statistically significant. Less consistent associations with HOMA-IR were observed for some of the remaining amino acids (Supplementary Table 1).

#### Sulfur amino acids

**Fig. 3:**
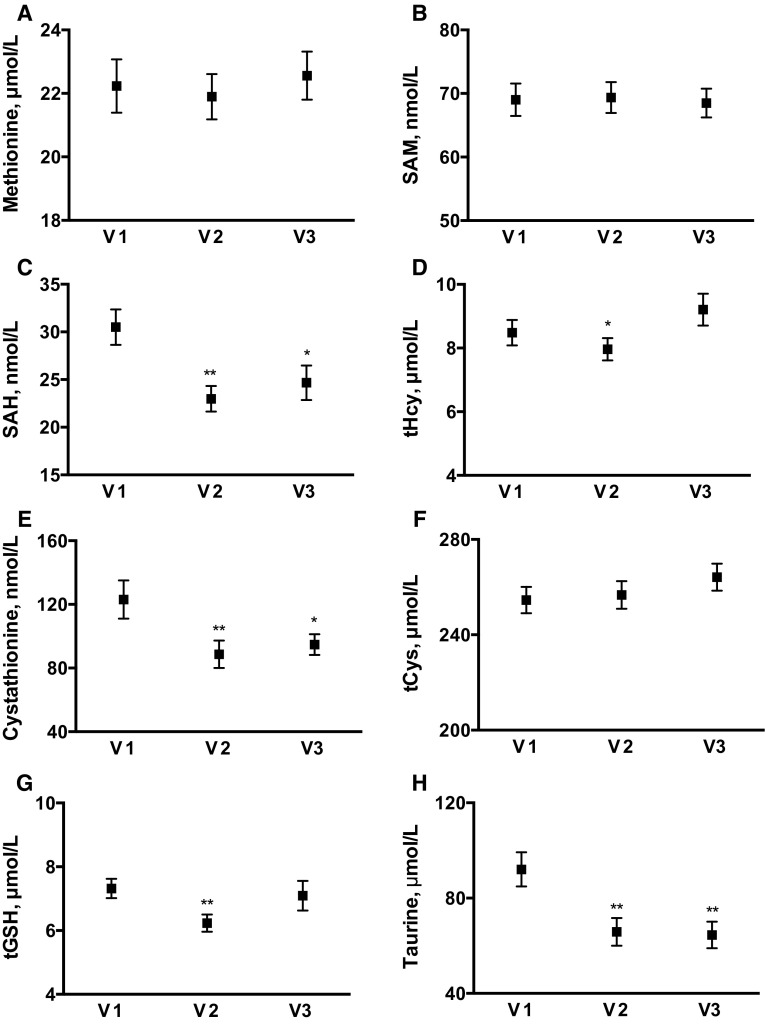
Fasting plasma sulfur amino acids and related metabolites at *V1* (baseline), *V2* (first week after diet change), and *V3* (last week of diet change). Data represent mean (SEM). *P < 0.05; **P ≤ 0.004 for pairwise comparisons versus V1, with repeated measures ANOVA *P* < 0.05

The changes in plasma SAAs and related metabolites were less consistent (Fig. 3). The diet change did not affect either methionine or cysteine, the two proteinogenic SAAs that are ingested in the diet. However, plasma tCys appears to have been maintained at the expense of downstream compounds. Plasma taurine decreased from 92 ± 7 μmol/L to 66 ± 6 (*P* = 0.003) and 65 ± 6 μmol/L (*P* = 0.003) at V2 and V3, respectively. A dip in tGSH was also observed at V2, but levels were restored to baseline levels by V3. Plasma cystathionine was consistently decreased at both V2 and V3 (by 28 and 23 %, respectively).

#### Other amino acids

**Fig. 4:**
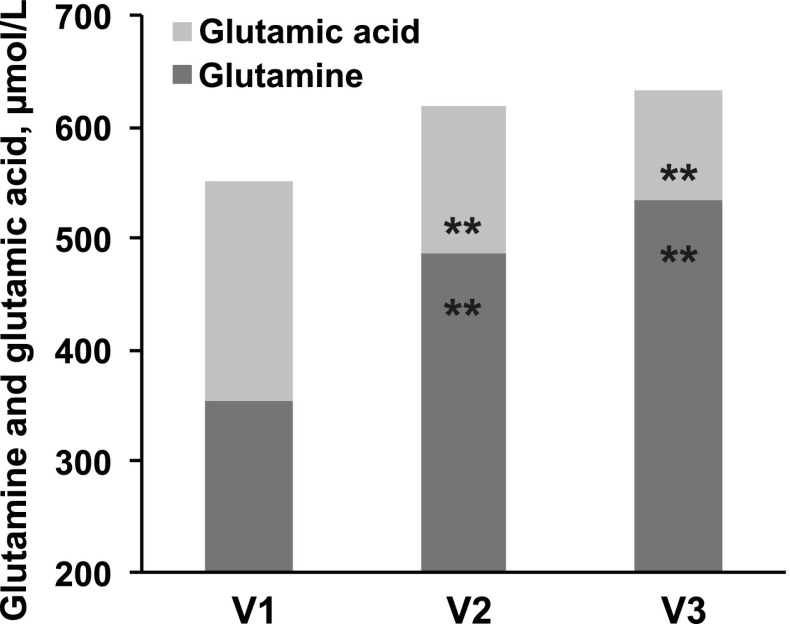
Changes in glutamine and glutamic acid. Data represent mean. **P < 0.001 for pairwise comparisons versus V1, with repeated measures ANOVA *P* < 0.05

The quantitatively largest changes were observed in glutamic acid and glutamine. The sum of both increased from 550 ± 13 to 619 ± 13 and 635 ± 14 μmol/L at V2 and V3, respectively (both *P* < 0.001 vs. V1). However, this increase was due to an increase in glutamine at the expense of glutamic acid (Fig. 4). Glutamic acid concentrations at V2 and V3 declined to 72 and 52 % of baseline values, whereas glutamine showed a 49 % increase at V3 relative to V1. No consistent changes were observed in plasma serine, ornithine, or proline, while plasma arginine showed a progressive increase that was significant only at V3 (Supplementary Figure 1).

## Discussion

The increasing recognition that BCAAs and SAAs are associated with adiposity and insulin resistance raises interest in identifying the determinants of their plasma levels in humans. Both high intake of red meat [1–3] and elevated plasma BCAAs [12–14] are independently associated with adiposity and predict future insulin resistance and type 2 diabetes. Here we show that abstinence from red meat and other sources of animal protein apart from fish decreased plasma BCAAs, without altering insulin sensitivity. On the other hand, the SAA cysteine, which strongly correlates with fat mass and insulin resistance [16, 17], and its precursors methionine and SAM were less responsive to dietary modification. Our data point to the importance of  habitual diet in determining fasting plasma BCAA concentrations.

The qualitative change of diet without limiting caloric intake in the different religious fasts [23] provides an opportunity to study the acute metabolic/metabolomic response to adopting different diets, without confounding the associated weight loss- and health-related interventions. To our knowledge, this is the first such study of an Egyptian Orthodox fast, but some metabolic effects have been previously reported in the essentially similar Greek Orthodox fast [21]. A fairly consistent finding is decreased total cholesterol and LDL cholesterol [21, 24], as observed in the present study.

The observed decline of plasma BCAA and aromatic amino acid concentrations is of interest in the context of their association with insulin resistance and type 2 diabetes. In the present study, plasma BCAA concentrations correlated positively with HOMA-IR in the two visits following the diet change. The elevation of BCAA in obesity and insulin resistance was first reported nearly half a century ago [25]. These findings were recently revived and extended by observations that BCAAs consistently emerge in non-targeted metabolomics as predictors of future diabetes and insulin resistance [13, 14], prompting some to hypothesize a causal role for BCAAs in these conditions. As reviewed by Lynch and Adams [26], some researchers have postulated that persistent activation of mammalian target of rapamycin complex 1 signalling in response to BCAA elevation inhibits insulin signal transduction. However, this view is challenged by recent data showing that short-term exposure to elevated plasma BCAA did not impair insulin sensitivity [26].

BCAA elevation might alternatively result from the insulin-resistant state, which is linked to reduced expression/action of BCAA catabolic enzymes (reviewed in [27, 28]). Bariatric surgery-induced weight loss was associated with up-regulation of these enzymes in human adipose tissue and decreased plasma BCAAs [19]. Further, insulin-sensitizing therapy in diabetic individuals lowered plasma concentrations of several BCAAs and aromatic amino acids [29]. However, the evidence is not consistent, since weight loss and enhanced insulin sensitivity brought about by calorie restriction often failed to lower BCAAs [30, 31]. In our young, insulin-sensitive study population, the decline in plasma BCAAs with restriction of animal protein occurred in the absence of changes in fat mass or HOMA-IR. This suggests that the BCAA reduction was not determined by insulin sensitivity, but by the change in dietary protein quality.

The non-fasting plasma amino acid profile of vegans, vegetarians, predominant fish eaters, and meat-eaters was recently reported [32]. Correlations of dietary intake with plasma levels were noted for leucine, isoleucine, valine, tryptophan, tyrosine, and methionine, but not for glutamate or phenylalanine. Leucine, valine, tyrosine, and tryptophan were lower in vegans compared to other groups [32]. In Mexican women, a meal containing animal protein elicited clearly larger increments in plasma BCAAs and aromatic amino acids over a 4-h period postprandially compared to a vegan meal [33]. Administering controlled high-meat or high-milk diets to children for 7 days also raised overnight-fasted plasma BCAAs [8]. The decline in BCAAs in the present study in response to decreased animal protein intake thus extends epidemiologic [32], and acute interventional data [8, 33], by demonstrating an effect of short-term modification of habitual diet on BCAAs. Our use of overnight-fasted samples precludes the possibility that plasma BCAA directly reflect recent dietary intake. Changes in tissue BCAA metabolism [34] and/or in gut microbiome [35] in response to altered dietary protein may have played a role in the observed changes in circulating levels.

The small decrease in BMI observed in the current study was noted in some [21, 24], but not all [36], similar studies. The present study showed that the decrease in BMI was mediated by a decline in lean mass (by an average of 0.7 kg) by the end of the 6-week fast. The drop in plasma essential amino acid concentrations, including BCAAs, may explain the decline in lean mass. Circulating essential amino acids were shown to exert a major influence on muscle protein synthesis in humans, independent of muscle amino acid availability [37]. Consumption of a serving of lean beef triggered an increase in plasma leucine that peaked ~100 min after the meal and acutely enhanced skeletal muscle protein synthesis [38].

In contrast to BCAA, plasma SAA did not show a response to the diet change. Cysteine and methionine are more abundant in animal proteins than in plant proteins [20]. In 570 elderly subjects, habitual intake of proteins from animal and plant sources showed positive and inverse associations, respectively, with plasma tCys and SAM, but not methionine [15]. tCys was associated particularly with dairy intake, while SAM correlated with meat consumption. Conflictingly, in 812 women, plasma tCys was unrelated to cystine or methionine intakes [39]. In view of these discrepant findings, it was of interest to determine whether short-term abstinence from most animal-derived food might lower tCys and SAM concentrations.

Plasma methionine, SAM, and tCys remained stable. One possible explanation is that the decrease in methionine intake was not sufficient to lower plasma levels, in contrast to a strict vegan diet [32]. It is also possible that compensatory changes in SAA metabolism occur to maintain the levels of key metabolites. Plasma cystathionine shows greater variation in response to intake of methionine than plasma methionine [40]. The consistent decrease in cystathionine in the present study suggests reduced flux through the transsulfuration pathway to conserve methionine [41]. Cysteine dioxygenase (CDO) plays a central role in maintaining cysteine levels by regulating the conversion of cysteine to taurine [42]. Feeding excess cystine to rodents had no effect on plasma tCys, but increased taurine [43]. In the present study, a marked drop in plasma taurine occurred despite increased fish intake, which is a positive determinant of taurine levels [43], suggesting suppression of the CDO pathway.

The study benefits from several strengths, including high compliance, fasting measurements assessing both acute and longer-term amino acid changes, and lack of the confounding associated with health-related diet interventions. However, it is limited by the small size. Another weakness is the lack of quantitation of dietary intakes, which may have revealed changes in macronutrient intakes, further explaining the data. We thus hope that our findings will stimulate larger studies of the effects of diet quality on the amino acid profile.

## Conclusion

In summary, we show that changing dietary protein sources toward fish- and plant-based sources in an ad libitum setting lowers the plasma BCAA that have been linked to diabetes risk. The effect is already apparent within 1 week of changing the diet. Our findings suggest that BCAA elevation in humans may be a marker for dietary patterns that are associated with diabetes and obesity, such as excess intake of red meat, and that plasma BCAA profile is amenable to dietary modification in humans.

## Electronic supplementary material

Below is the link to the electronic supplementary material.

Supplementary Fig. 1 Fasting plasma amino acids at V1 (baseline), V2 (first week after diet change), and V3 (last week of diet change). Data represent mean (SEM). *P < 0.05 for pairwise comparisons vs V1, with repeated measures ANOVA *P* < 0.05 (EPS 85 kb)

Supplementary material - Methods (DOCX 100 kb)

Supplementary Table 1 (DOCX 78 kb)
