# Supplementary material for: Amino acid changes during transition to a vegan diet supplemented with fish in healthy humans
Source: Eur J Nutr. 2016 Jun 11;56(5):1953–62. doi: 10.1007/s00394-016-1237-6 (PMC5534203; doi:10.1007/s00394-016-1237-6)
Supplement: Supplementary file 2 — Supplementary material - Methods (DOCX 100 kb) [file 394_2016_1237_MOESM2_ESM.docx]

**Supplementary Method**

*LC-MS/MS analysis of taurine, serine, glutamine, glutamic acid, and creatinine*

Taurine, serine, glutamine, glutamic acid, and creatinine were extracted from 10 µL plasma by adding an equal volume of a solution containing isotopically labelled internal standards (Cambridge Isotopes Laboratories, Andover, MA, US), and 100 µL methanol. Samples were mixed thoroughly and precipitated at -80°C for 5 min, followed by centrifugation at 4’000 rpm for 15 min at 8°C. 20 µL supernatant was added to 80 µL diluent (Mobile phase A; 0.5 % formic acid and 0.3 % heptafluorobutyric acid (HFBA) in water), and 15 μL was injected onto a Kinetex Core Shell C18 column (100 x 4.6 mm, 2.6 μm; Phenomenex, Torrance, CA, US) kept at 25°C. Gradient elution at 800 µL/min with mobile phases A and B (0.5 % formic acid and 0.3 % HFBA in acetonitrile) was used. 0 % B was initially held at for 2.0 min, followed by a linear gradient reaching 60 % B at 4.5 min, and held there for 1.5 min. The column was equilibrated at 0 % B for 0.5 min before the next injection, for a total run time of 6.5 min. Gas 1 and 2 were set at 60, collision gas at medium, and the curtain gas, ion spray voltage, and source temperature were set at 45, 3500 V, and 650°C, respectively. Compound-dependent parameters for all analytes and internal standards can be found in Supplementary Table 1. Optimisation of MRM transitions, data acquisition, and analysis were performed with Analyst 1.6.1 (AB Sciex, Framingham, MA, US).

**Supplementary Table 1** Compound-dependent parameters used for all analytes and their respective internal standard.

| Analyte | Q1 (Da)^1^ | Q3 (Da) | RT (min) | DP (V) | EP (V) | CE (V) | CXP (V) |
| --- | --- | --- | --- | --- | --- | --- | --- |
| Taurine | 125.9 | 108 | 1.2 | 36 | 10 | 17 | 10 |
| Tarine-d_4_ | 129.9 | 112 | 1.2 | 36 | 10 | 15 | 10 |
| Serine | 106 | 60 | 1.8 | 50 | 10 | 16 | 10 |
| Serine-d_3_ | 109 | 63 | 1.8 | 50 | 10 | 14 | 10 |
| Glutamine | 146.9 | 130 | 2.1 | 36 | 8 | 27 | 8 |
| Glutamine-d_5_ | 152 | 135 | 2.1 | 36 | 8 | 24 | 8 |
| Glutamic acid | 148.1 | 102 | 2.4 | 36 | 10 | 9 | 6 |
| Glutamic acid-d_3_ | 151.1 | 105 | 2.4 | 36 | 10 | 15 | 6 |
| Creatinine | 114 | 86 | 4.0 | 56 | 10 | 15 | 10 |
| Creatinine-d3 | 117 | 89 | 4.0 | 56 | 10 | 15 | 10 |

^1^ CE, collision energy; CXP, cell exit potential; DP, declustering potential; EP, entrance potential; Q1, parent ion; Q3, daughter ion; RT, retention time.
