# Supplementary material for: Amino acid changes during transition to a vegan diet supplemented with fish in healthy humans
Source: Eur J Nutr. 2016 Jun 11;56(5):1953–62. doi: 10.1007/s00394-016-1237-6 (PMC5534203; doi:10.1007/s00394-016-1237-6)
Supplement: Supplementary file 3 — Supplementary Table 1 (DOCX 78 kb) [file 394_2016_1237_MOESM3_ESM.docx]

|  | **HOMA-IR** | | |
| --- | --- | --- | --- |
|  | **V1** | **V2** | **V3** |
| Arginine | -0.29 | -0.04 | -0.14 |
| **Cystathionine** | 0.10 | **0.45*** | 0.36 |
| Glutamic acid | -0.22 | 0.09 | 0.06 |
| Glutamine | 0.25 | 0.40 | 0.22 |
| **Glutamine/glutamic acid** | 0.21 | **0.45*** | 0.25 |
| **Isoleucine** | 0.27 | **0.46*** | **0.53*** |
| **Leucine** | 0.25 | **0.43*** | **0.48*** |
| Methionine | 0.06 | 0.28 | 0.29 |
| **Ornithine** | -0.10 | 0.20 | **0.54*** |
| Phenylalanine | 0.30 | 0.29 | 0.19 |
| Proline | 0.13 | 0.31 | 0.27 |
| S-adenosylhomocysteine | -0.22 | 0.16 | 0.17 |
| **S-adenosylmethionine** | 0.07 | **0.62*** | 0.08 |
| Serine | -0.28 | -0.29 | -0.08 |
| Taurine | -0.29 | -0.11 | -0.24 |
| **Total cysteine** | -0.02 | **0.61*** | 0.01 |
| Total glutathione | -0.22 | 0.02 | -0.22 |
| Total homocysteine | 0.02 | 0.18 | 0.30 |
| Tryptophan | 0.23 | -0.05 | 0.09 |
| **Tyrosine** | -0.02 | **0.42*** | 0.34 |
| **Valine** | 0.26 | **0.46*** | **0.50*** |

**Supplementary Table 1. Correlations of fasting plasma amino acids with HOMA-IR at the 3 timepoints ^1^.**

(1) Spearman correlation coefficients before the diet change (V1), and one (V2) and 6 (V3) weeks into the diet change. * P<0.05. Bold font is used for amino acids showing significant (P <0.05) correlations with HOMA-IR at any time-point. N = 27. HOMA-IR, homeostasis model of insulin resistance.
